# A third copy of the Down syndrome cell adhesion molecule (*Dscam*) causes synaptic and locomotor dysfunction in *Drosophila*

**DOI:** 10.1016/j.nbd.2017.11.013

**Published:** 2018-02

**Authors:** Simon A. Lowe, James J.L. Hodge, Maria M. Usowicz

**Affiliations:** School of Physiology, Pharmacology and Neuroscience, University of Bristol, University Walk, Bristol BS8 1TD, UK

**Keywords:** DS, Down syndrome, HSA21, human chromosome 21, *DSCAM*, Down syndrome cell adhesion molecule, NMJ, neuromuscular junction, EJP, excitatory junction potential, mEJP, miniature excitatory junction potential, Down syndrome, DSCAM, *Drosophila*, Synaptic transmission, Locomotion

## Abstract

Down syndrome (DS) is caused by triplication of chromosome 21 (HSA21). It is characterised by intellectual disability and impaired motor coordination that arise from changes in brain volume, structure and function. However, the contribution of each HSA21 gene to these various phenotypes and to the causal alterations in neuronal and synaptic structure and function are largely unknown. Here we have investigated the effect of overexpression of the HSA21 gene *DSCAM* (Down syndrome cell adhesion molecule), on glutamatergic synaptic transmission and motor coordination, using *Drosophila* expressing three copies of *Dscam1*. Electrophysiological recordings of miniature and evoked excitatory junction potentials at the glutamatergic neuromuscular junction of *Drosophila* larvae showed that the extra copy of *Dscam1* changed the properties of spontaneous and electrically-evoked transmitter release and strengthened short-term synaptic depression during high-frequency firing of the motor nerve. Behavioural analyses uncovered impaired locomotor coordination despite preserved gross motor function. This work identifies *DSCAM* as a candidate causative gene in DS that is sufficient to modify synaptic transmission and synaptic plasticity and cause a DS behavioural phenotype.

## Highlights

• *Drosophila* expressing a third copy of *Dscam* have altered neuromuscular transmission.
• *Drosophila* expressing a third copy of *Dscam* have deficits in locomotor coordination.
• *Drosophila* are a powerful system for studying single-gene effects in Down syndrome.

## 1 Introduction

Down syndrome (DS, also known as Down's syndrome) is caused by triplication of all or part of human chromosome 21 (HSA21; trisomy 21, OMIM ID: 190685) (Antonarakis, 2016) and occurs in 1 in ~ 900 live births (de Graaf et al., 2017, Wu and Morris, 2013). DS is characterised by mild to severe intellectual disability (Lott and Dierssen, 2010), motor impairments (Malak et al., 2013, Rao et al., 2017), and early onset dementia that is a form of Alzheimer's disease (Ballard et al., 2016, Hithersay et al., 2017). These clinical features are accompanied by changes in the number and structure of brain neurons and synapses, as well as changes in the concentrations of the neurotransmitters, GABA and glutamate, and their receptors (Contestabile et al., 2017, Dierssen, 2012, Rueda et al., 2012). The cognitive and motor deficits in DS arise from aberrant information processing in the brain that is due, in part, to changes in synaptic transmission or synaptic plasticity. Individuals with DS have impaired synaptic plasticity in the motor cortex (Battaglia et al., 2008). In cultured neurons derived from trisomy 21 induced pluripotent stem cells, the extra copy of HSA21 results in fewer excitatory and inhibitory connections, as well as a decrease in the frequency of spontaneous excitatory and inhibitory postsynaptic currents (sEPSCs and sIPSCs) (Weick et al., 2013).

How synaptic transmission and plasticity are changed in the brain by triplication of HSA21 genes has also been investigated in a variety of mouse models of DS that express a third copy of different numbers of HSA21 mouse orthologues (including Ts65Dn, Ts1Rhr, Ts1Cje, Ts2Cje, Ts1Yah, Dp(16)1Yey/+, Dp(10)1Yey/+;Dp(16)1Yey/+;Dp(17)1Yey/+) or a human chromosome 21 (Tc1). These electrophysiological studies have revealed alterations in basal synaptic transmission at some brain synapses. In cultured hippocampal neurons, miniature EPSCs (mEPSCs) have a faster rise and decay (Best et al., 2008). In CA3 neurons in hippocampal slices, there is a decrease in the frequency of both miniature IPSCs (mIPSCs) and mEPSCs (Hanson et al., 2007, Stagni et al., 2013) alongside a decrease in mIPSC amplitude and no change in long term potentiation (LTP) of evoked excitatory transmission (Hanson et al., 2007). Likewise, in neocortical neurons, sIPSCs and sEPSCs are less frequent but sEPSC amplitudes are reduced (Cramer et al., 2015). In cerebellar slices, evoked EPSCs at parallel fibre-Purkinje cell synapses, but not at climbing fibre-Purkinje cell synapses, are slower (Galante et al., 2009) or unchanged (Das et al., 2013), and long-term depression (LTD) at parallel fibre-Purkinje cell synapses is unaltered (Das et al., 2013, Galante et al., 2009). The probability of glutamate release from cerebellar granule cells is enhanced (Das et al., 2013) and these neurons receive weaker tonic GABAergic inhibition (Szemes et al., 2013). Other studies have identified alterations in synaptic plasticity at some excitatory glutamatergic brain synapses. LTP and LTD in striatal spiny neurons are unaffected, but LTP is decreased in striatal cholinergic interneurons (Di Filippo et al., 2010). Extracellular recordings of field EPSPs report weaker LTP but stronger LTD at excitatory synapses on CA1 neurons, due to enhanced GABAergic transmission (Andrade-Talavera et al., 2015, Belichenko et al., 2007, Chakrabarti et al., 2010, Costa and Grybko, 2005, Das et al., 2013, Deidda et al., 2015, Kaur et al., 2014, Mitra et al., 2012, Olson et al., 2007, Pereira et al., 2009, Siarey et al., 1999, Siarey et al., 1997, Siarey et al., 2005, Yu et al., 2010a, Yu et al., 2010b, Zhang et al., 2014), which may include an increase in excitatory, as well as inhibitory, GABAergic signalling mediated by GABA_A_ or GABA_B_ receptors (Contestabile et al., 2017, Deidda et al., 2015). Extracellular recordings of field EPSPs also report impaired LTP in synapses of the dentate gyrus (DG) and perirhinal cortex, due to enhanced GABAergic transmission (Belichenko et al., 2009, Belichenko et al., 2015, Contestabile et al., 2013, Fernandez et al., 2007, Kleschevnikov et al., 2012, Kleschevnikov et al., 2004, Morice et al., 2008, O'Doherty et al., 2005, Roncace et al., 2017). Although weaker LTP at hippocampal CA1 and DG synapses has largely been ascribed to enhanced GABAergic signalling (Contestabile et al., 2017), some studies report compromised glutamate release in response to closely spaced pairs or trains of stimuli at DG synapses (Kaur et al., 2014) and CA1 synapses (Andrade-Talavera et al., 2015, Siarey et al., 2005), that may contribute to the weaker LTP.

Altogether, the electrophysiological studies indicate that synaptic dysfunction in DS is not the same at all brain synapses, and both glutamatergic and GABAergic transmission can be altered. The contribution of individual HSA21 genes to the changes in synaptic function in DS is incompletely understood (Gupta et al., 2016). One way to explore this gene-phenotype relationship, that is faster and less costly than using mouse models, is to overexpress individual orthologous genes in *Drosophila* and examine their consequences (Chang and Min, 2009, Cvetkovska et al., 2013). *Drosophila* is an established model of genetic disorders due to its short lifespan, well defined neural circuits, genetic tractability, conservation of molecular mechanisms driving cellular and physiological processes, and the existence of *Drosophila* orthologues for ~ 75% of disease causing human genes (Androschuk et al., 2015, Perrimon et al., 2016, Ugur et al., 2016). One candidate HSA21 gene for such a study is Down syndrome cell adhesion molecule (*DSCAM*).

*DSCAM* is a member of the immunoglobulin superfamily with four *Drosophila* homologues, *Dscam1*–*4* (Tadros et al., 2016), of which *Dscam1* is the prototypical member and the most studied. In both *Drosophila* and mice, *Dscam* is highly expressed throughout the central and peripheral nervous system (Barlow et al., 2002, Barlow et al., 2001, Wang et al., 2004), in a highly regulated spatiotemporal pattern (Saito et al., 2000, Yamakawa et al., 1998), and is locally translated in dendrites and growth cones (Alves-Sampaio et al., 2010, Jain and Welshhans, 2016). Loss of function studies have uncovered a vital role for DCSAM in the structural development of the nervous system that is conserved from *Drosophila* to mammals. It is necessary for self-avoidance during neurite outgrowth (de Wit and Ghosh, 2016, Fuerst et al., 2009, Hutchinson et al., 2014), normal dendritic branching and spine formation (de Andrade et al., 2014, Maynard and Stein, 2012, Zhang et al., 2015, Zhu et al., 2006), correct axon targeting (Cvetkovska et al., 2013, Hutchinson et al., 2014, Liu et al., 2009) and the formation of synapses (Hummel et al., 2003, Li et al., 2009, Millard et al., 2010). *Dscam* also regulates clustering of postsynaptic AMPA-like ionotropic glutamate receptors in *Aplysia* neurons (Li et al., 2009), and is essential for the correct operation of locomotor and sensorimotor circuits that underpin locomotor coordination and motor learning in mice (Lemieux et al., 2016, Thiry et al., 2016, Xu et al., 2011). Overexpression of murine *Dscam* in mice increases cell death and disrupts dendrite targeting in the retina (Li et al., 2015), promotes axonal growth of retinal ganglion cells (Bruce et al., 2017) and disrupts dendritic and axonal branching in mouse cultured hippocampal or cortical neurons (Alves-Sampaio et al., 2010, Jain and Welshhans, 2016). Overexpression of *Dscam* in *Drosophila* causes abnormal branching of sensory axons and impaired transfer of information along the neural circuit mediating touch perception (Cvetkovska et al., 2013) and enlargement of presynaptic terminals of sensory neurons in *Drosophila* larvae (Kim et al., 2013). Altogether, these studies indicate that the effects of *DSCAM* are gene dosage sensitive.

The extra copy of HSA21 in DS causes overexpression of *DSCAM* in the brain from childhood to adulthood, particularly in cerebral cortical neurons, cerebellar Purkinje cells and fibres in the cerebellar granule layer (Saito et al., 2000). *Dscam* overexpression is replicated in hippocampal and cerebral neurons, and in the whole-brain of mouse models of DS (Alves-Sampaio et al., 2010, Amano et al., 2004, Guedj et al., 2015, Perez-Nunez et al., 2016), wherein it disrupts dendritic growth (Perez-Nunez et al., 2016). *Dscam* is also overexpressed in mice that overexpress another HSA21 gene orthologue *App*, which encodes the amyloid precursor protein (Jia et al., 2011) and is a causative gene in cognitive dysfunction in DS and Alzheimer's Disease (Wiseman et al., 2015). Dosage sensitivity of the effects of *DSCAM* in DS is supported by the ability of *Dscam* loss of function mutations to correct the disrupted dendritic fasciculation of a subset of retinal ganglion cells in the Ts65Dn mouse model of DS (which has three copies of a chromosomal segment orthologous to a HSA21 segment that contains *DSCAM*) (Blank et al., 2011). In contrast, this normalisation of *Dscam* copy number did not correct the enhanced ipsi/contralateral segregation of retinogeniculate projections observed in Ts65Dn mice, suggesting an essential role for other triplicated genes in this phenotype (Blank et al., 2011).

As outlined above, many studies have shown that *DSCAM* shapes dendritic, axonal and synaptic structure in a dose-dependent manner; many of the dose-dependent physical changes predict changes in synaptic communication. The elevated *DSCAM* expression in DS cerebellar Purkinje cells and fibres in the cerebellar granule layer (Saito et al., 2000) suggests a role in motor deficits in DS; the fibres convey information to the Purkinje cells, which integrate the information and carry signals out of the cerebellar cortex to direct motor planning, execution and coordination (Apps and Garwicz, 2005). As there have been no direct studies of the effects of *DSCAM* overexpression on synaptic transmission or plasticity or motor function, we investigated the effects of a third copy of *DSCAM* on glutamatergic synaptic transmission and locomotor function. We took advantage of a previously described *Drosophila* model that expresses a third copy of *Dscam1* (hereafter *Dscamx3*) under its endogenous promoter and has elevated levels of *Dscam1* mRNA and *Dscam1* protein (Cvetkovska et al., 2013). Synaptic transmission and short-term plasticity were examined at the larval NMJ, a glutamatergic synapse that is considered an excellent model of mammalian central glutamatergic synapses and is readily accessible for electrophysiological recording (Harris and Littleton, 2015, Menon et al., 2013). Locomotor function of larvae was assessed in two behavioural assays.

## 2 Materials and methods

### 2.1 *Drosophila* strains

Flies were raised with a 12 h:12 h light dark (LD) cycle with lights on at ZT 0 (Zeitgeber Time) on standard *Drosophila* medium (0.7% agar, 1.0% soya flour, 8.0% polenta/maize, 1.8% yeast, 8.0% malt extract, 4.0% molasses, 0.8% propionic acid, 2.3% nipagen) at 25 °C and collected ~ 6 days after egg laying. *Canton Special white*- (*CSw*-), from Dr. Scott Waddell (University of Oxford) were used as a control. Flies with three copies of *Dscam* (*Dscamx3*) were generated by crossing homozygous *Dscam1*^*BAC*^ flies (Cvetkovska et al., 2013), which contain an extra copy of the full length of the *Dscam1* gene in a bacterial artificial chromosome, to *CSw*- flies, to generate heterozygous offspring containing one extra copy. The *Dscam1*^*BAC*^ flies were kindly donated by Dr. Brian Chen (McGill University Health Centre) and have previously been verified to overexpress *Dscam1* mRNA and *Dscam1* protein (Cvetkovska et al., 2013).

### 2.2 Behaviour

For the free-crawling assay, a single third instar wandering larva was washed in a drop of distilled H_2_O, transferred to a 9.5 cm dish containing 1.6% agarose and allowed 30 s to acclimatise. The dish was placed over a 0.5 cm grid and the number of lines the larva crossed in one minute was counted. For the self-righting assay, a larva was gently rolled onto its back using a fine distilled H_2_O-moistened paintbrush and the time for it to right itself was recorded (Park et al., 2002). Experiments took place at 25 °C.

### 2.3 Antibody staining and visualisation

Wandering third instar larvae were dissected in ice-cold, Ca^2 +^-free HL3.1-like solution (in mM: 70 NaCl, 5 KCl, 10 NaHCO_3_, 115 sucrose, 5 trehalose, 5 HEPES, 10 MgCl_2_) to produce a larval “fillet” (Brent et al., 2009). The fillet was fixed for 30 min in 4% paraformaldehyde (Sigma Labs) and washed three times in 1% Triton-X (Sigma Labs), then blocked for one hour in 5% normal goat serum (Fitzgerald Industries) and 1% Triton-X at room temperature. It was incubated overnight in 1:500 FITC-conjugated anti-horseradish peroxidase (HRP-FITC) (Jackson Immunoresearch Laboratories) and 1:500 mouse anti-Discs large (Dlg) primary antibody (Sherwood et al., 2004), then for two hours in 1:500 AlexaFluor 633-conjugated goat anti-mouse secondary antibody at room temperature. Fillets were washed and mounted on a coverslip in Vectashield (Vector Laboratories). *Z*-series of larval NMJs were imaged on a Leica SP5-II confocal laser-scanning microscope using an oil immersion 40 × objective. The number of boutons at the NMJ of muscle 6/7 in segment A2 was counted manually. Satellite boutons were identified as a single bouton with 3 or more boutons budding from it (Menon et al., 2013). ImageJ (rsb.info.nih.gov/ij/) was used to manually outline muscles 6 and 7 and hence calculate their area.

### 2.4 Electrophysiology

Wandering third instar larvae were dissected in ice-cold, Ca^2 +^-free HL3.1-like solution, as for antibody staining, then the motor nerves were severed below the ventral ganglion and the brain was removed. CaCl_2_ (1 mM) was added to the bath solution for intracellular recording from muscle 6 of abdominal segments 2–4. Sharp microelectrodes (thick-walled borosilicate glass capillaries, pulled on a Sutter Flaming/Brown P-97 micropipette puller) were filled with 3 M KCl and had resistances of 20–30 MΩ. For recording of stimulus evoked excitatory junction potentials (EJPs), severed nerves were drawn into a thin-walled glass-stimulating pipette and stimulated 10 times with square-wave voltage pulses (0.1 ms, 10 V, A-M Systems Model 2100 Isolated Pulse Simulator) at a frequency of 0.1 Hz.

EJPs and spontaneously-occurring miniature EJPs (mEJPs) were recorded from muscle 6, segments A2–4, at a controlled room temperature of 22–25 °C with a Geneclamp 500 amplifier (Axon Instruments) and were further amplified with a LHBF-48 × amplifier (NPI Electronic). The membrane potential was set to − 70 mV with current input at the start of the recording. Voltage signals were low-pass filtered at 1.67 kHz (10 kHz 4 pole Bessel on Geneclamp 500, 1.7 kHz 8-pole Bessel on LHBF-48x) and digitised at 25 kHz by a CED-1401 plus A/D interface (Cambridge Electronic Design, UK) using Spike2 software (v. 5.13) (CED, Cambridge, UK). Synaptic potentials were analysed off line using Strathclyde Electrophysiology Software WinEDR (v3.5.2) and GraphPad Prism (v.6). All synaptic events were verified manually.

Recordings were discarded if the initial resting membrane potential was more positive than − 60 mV or varied by > 10% during recording for at least 20 min. The input resistance of the muscle was measured by injecting current using the Axon Geneclamp 500, to bring the membrane potential to − 100, − 80, − 60 and − 40 mV and subtracting the electrode resistance from the slope of the resulting voltage/current graph. There was no difference between control and *Dscamx3* larval NMJs in mean resting membrane potential (*CSw*-, − 69.00 ± 2.33 mV, *N* = 8; *Dscamx3*, − 71.38 ± 1.87 mV, *N* = 8; *P* = 0.439) or input resistance (*CSw*-, 3.16 ± 0.64 MΩ, *N* = 8; *Dscamx3*, 3.38 ± 0.7 MΩ, *N* = 8; *P* = 0.823).

The amplitudes and intervals of mEJPs were compared by creating a cumulative distribution for each genotype of 1600 measurements across 8 animals, with each animal contributing 200 values. To analyse the mEJP waveform, a mean mEJP was constructed for each recording from events that showed only a single clear peak and a smooth decay, which prevented distortion of the waveform by closely occurring mEJPs. A single exponential was fitted to the decay of the mean mEJP and the 10–90% rise-time was measured. Time zero for the exponential fit was set to the time at the peak of the mEJP. EJP waveforms were analysed by forming a mean EJP of 10 events, measuring the 10–90% rise-time of the mean event, and fitting the decay with the sum of three exponentials (time zero was set at the time of the peak). A mean weighted time constant of decay (τ_decay_) was calculated as A_1_.τ_1_ + A_2_.τ_2_ + A_3_.τ_3_, where A_1_, A_2_ and A_3_ are the fractional amplitudes of the three components, and τ_1_, τ_2_ and τ_3_ are their time constants.

For paired pulse analysis, two EJPs were evoked with stimuli separated by increasing intervals between 10 ms and 10 s. The second EJP was measured and the amplitude of the second event was expressed as fraction of the first. Pairs of stimuli were separated by 30 s. For high–frequency stimulation, trains of 10 EJPs were evoked at 10 Hz, 8 times at 1 min intervals. The amplitude of each event was expressed as a fraction of the mean amplitude of 10 single EJPs evoked at 0.1 Hz prior to the train (baseline). To investigate the effect of the stimulus trains on spontaneous release, 50 mEJPs per NMJ were analysed from immediately before and immediately after the trains. Amplitudes and inter-event intervals were measured and pooled from 8 NMJs to form cumulative amplitude distributions composed of 400 values.

### 2.5 Statistical analysis

Statistical analysis was conducted in GraphPad Prism (v. 6, La Jolla, CA). Data were tested for normality using the Kolmogorov-Smirnov test; where appropriate, means were compared using Student's unpaired *t*-test or medians were compared with a Mann-Whitney *U* test. Data are given as mean ± SEM (standard error of the mean) or median. *N* is the number of animals. Paired pulses and event trains were compared using repeated measures 2-way ANOVA. Cumulative distributions were compared with a Kolmogorov-Smirnov test. An α level of *P* < 0.05 was considered statistically significant.

## 3 Results

### 3.1 A third copy of *Dscam* altered basal spontaneous and evoked synaptic transmission

**Fig. 1:**
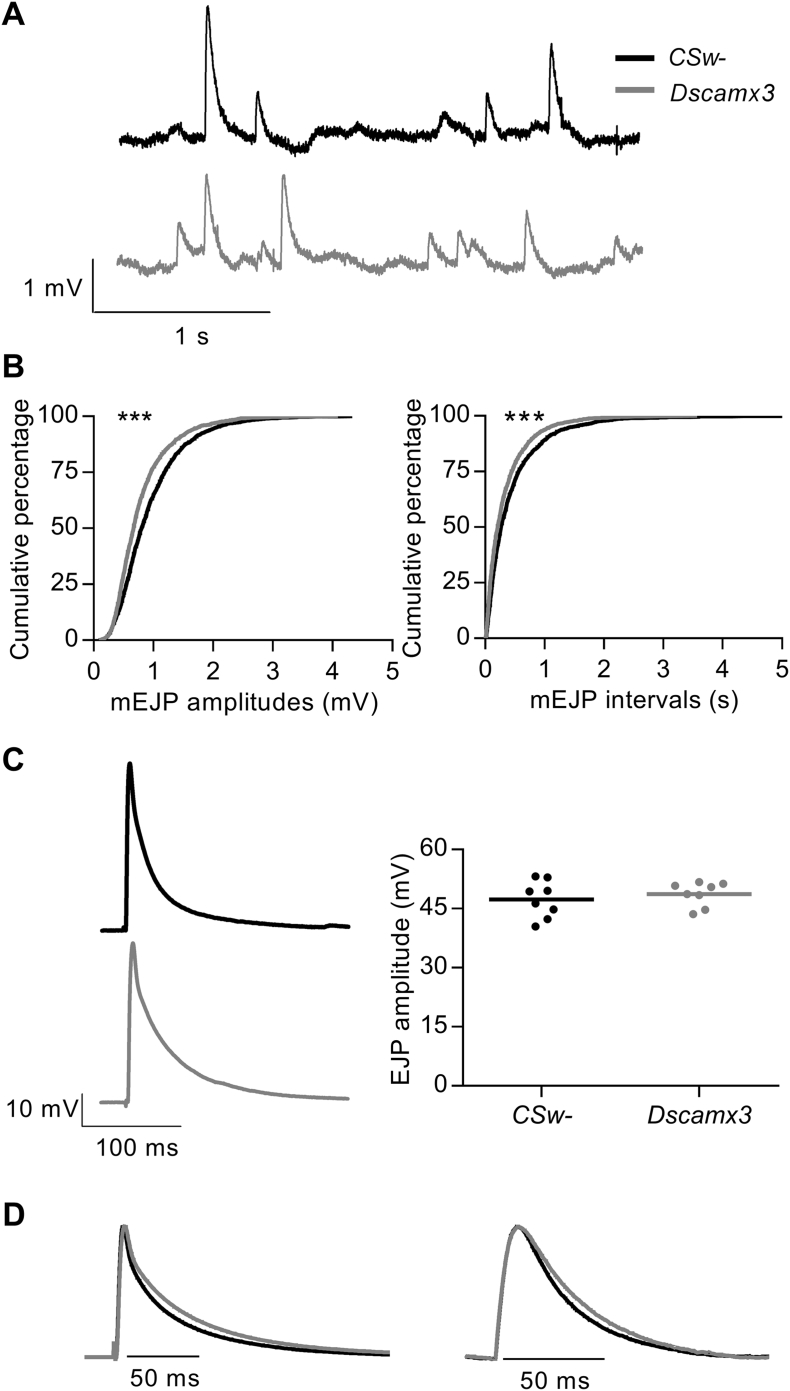
Basal synaptic transmission at *Dscamx3* larval NMJs. (A) Voltage recordings (3 s traces) from NMJs of *Drosophila* larvae expressing the normal two (*CSw*-, black) or three copies of *Dscam1* (grey) illustrating spontaneously occurring mEJPs at a membrane potential of − 70 mV. (B) Cumulative frequency distributions of mEJP amplitudes (left) and intervals (right) (1600 events in each distribution; 200 from each of 8 NMJs). mEJPs at *Dscamx3* NMJs were smaller (*P* < 0.0001) and more frequent (*P* < 0.0001). (C) (Left) Representative traces of a single nerve-evoked EJP at a *CSw*- NMJ and a *Dscamx3* NMJ. (Right) The mean EJP amplitude did not differ (*CSw*-, 47.37 ± 1.66 mV, *N* = 8; *Dscamx3*, 48.72 ± 1.08 mV, *N* = 8; *P* = 0.5086). (D) (Left) Average waveform of EJPs, formed from means of 10 events in 8 recordings. The mean rise time was slower for *Dscamx3* (*CSw*-, 2.27 ± 0.12 ms, *N* = 8; *Dscamx3* = 2.62 ± 0.08 ms, *N* = 8; *P* = 0.036), as was the decay (mean weighted τ_decay_ of three exponentials fitted to the decay; *CSw*-, 31.00 ± 1.74 ms, *N* = 8; *Dscamx3* = 38.60 ± 3.00 ms, *N* = 8; *P* = 0.0469). (Right) Average waveform of mEJPs, superimposed and normalised by the peak (grand average of mean mEJPs constructed from 50 to 100 events from each of 8 recordings). The mean rise time did not differ (*CSw*-, 6.24 ± 0.25 ms, *N* = 8; *Dscamx3*, 6.34 ± 0.52 ms, *N* = 8; *P* = 0.816). The decay was slower for *Dscamx3* (τ_decay_ of a single exponential fitted to the decay (not shown): *CSw*-, 27.0 ± 1.73 ms, *N* = 8; *Dscamx3*, 34.9 ± 1.74 ms, *N* = 8; *P* = 0.006).

The effect of a third copy of the *Dscam* gene on synaptic transmission at a glutamatergic synapse was examined by making intracellular microelectrode recordings at the NMJ of *Dscamx3 Drosophila* larvae (Fig. 1A). Spontaneously occurring miniature excitatory junction potentials (mEJPs) were smaller and more frequent than in control larvae (*CSw*-) containing 2 copies of *Dscam* (Fig. 1B). In contrast to the reduction in mEJP amplitude, the amplitudes of electrically-evoked, Ca^2 +^-dependent excitatory junction potentials (EJPs) were unaltered (Fig. 1C). Consequently, the approximate mean quantal content of EJPs (calculated as mean EJP amplitude/mean mEJP amplitude for each larva, not corrected for non-linear summation) was increased by 40% (*CSw*-, 47.23 ± 3.41, *N* = 8; *Dscamx3*, 66.64 ± 7.47, *N* = 8; *P* = 0.0331). Therefore, an extra copy of *Dscam* caused a decrease in the size of the postsynaptic depolarisations generated by single transmitter vesicles (the mEJPs). In parallel, there was an increase in the mean number of vesicles released by a nerve action potential which resulted in an unchanged EJP amplitude. In addition, there was a moderate lengthening of the decay of both mEJPs (by 29%) and EJPs (by 25%) (Fig. 1D). The cause of the slower decay was not investigated.

### 3.2 A third copy of *Dscam* enhanced depression during high frequency nerve stimulation

**Fig. 2:**
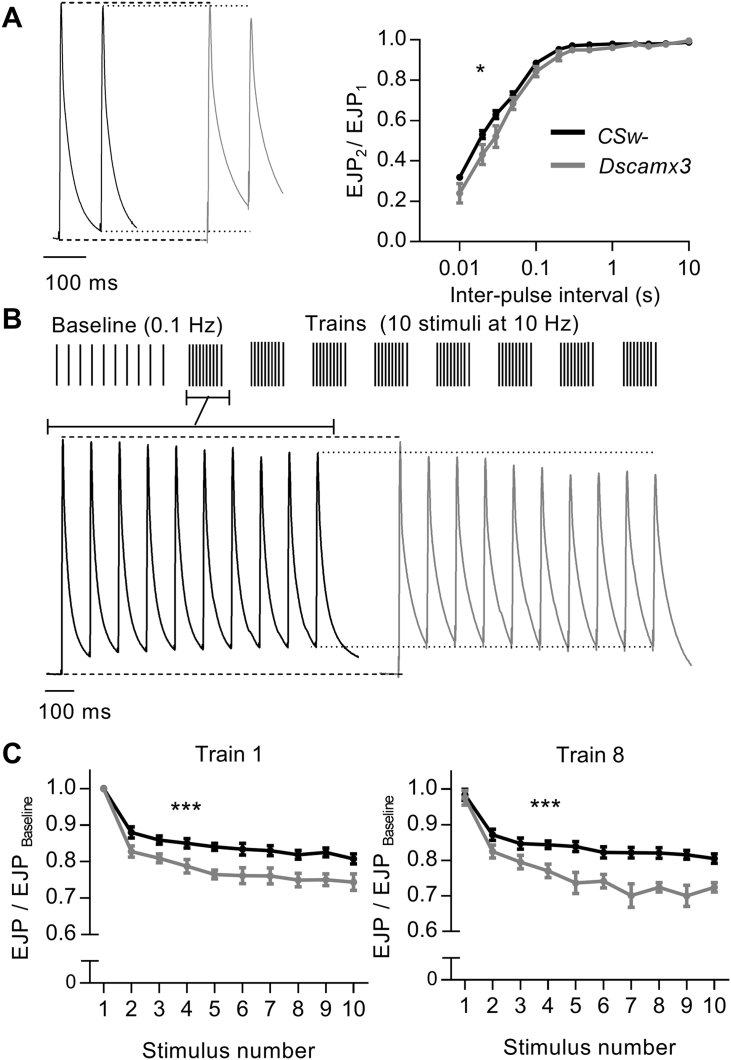
Synaptic depression is stronger at *Dscamx3* larval NMJs. (A) (Left) Representative pairs of stimulus evoked EJPs at control (*CSw*-, black) and *Dscamx3* (grey) NMJs. Dashed lines compare the first EJP (EJP_1_) and dotted lines compare the second EJP (EJP_2_). (Right) Plots of paired-pulse ratio (EJP_2_/EJP_1_) against inter-pulse interval reveal stronger depression at *Dscamx3* NMJs (*F* (8, 112) = 2.245, *P* = 0.0291). (B) High-frequency stimulation protocol. 10 EJPs were evoked at 0.1 Hz to establish a mean baseline amplitude followed by 8 trains of 10 EJPs at 10 Hz, at one minute intervals. Representative trains of EJPs recorded from *CSw*- and *Dscamx3* NMJs. Dashed lines compare EJP_1_ and dotted lines compare EJP_8_. (C) Plots of EJP amplitude during trains 1 (left) and 8 (right), expressed as a fraction of baseline amplitude (vertical bars represent SEM). The depression in EJP amplitude was greater at *Dscamx3* NMJs than at control NMJs during train 1 (*F* (9,126) = 3.98, *P* = 0.002) and train 8 (*F* (9,126) = 3.86, *P* = 0.0002). The depression in amplitude was constant for all trains within genotype (train 1 versus train 8: *CSw*- (*F* (9,126) = 0.42, *P* = 0.9229; *N* = 8); *Dscamx3* (*F* (9,126) = 1.23. *P* = 0.2834, *N* = 8).

To examine further the impact of a third copy of *Dscam* on electrically-triggered transmitter release, we compared synaptic plasticity at *Dscamx3* and control larval NMJs. In agreement with previous studies at control NMJs, nerve stimulation with pairs of pulses at varying intervals (between 10 ms and 10 s) caused depression of the amplitude of the second EJP relative to the first (Fig. 2A) (Dason et al., 2009, Gaviño et al., 2015, Kauwe and Isacoff, 2013). Such paired-pulse depression also occurred at *Dscamx3* NMJs, but it was more pronounced, particularly at intervals shorter than 300 ms (Fig. 2A). The strengthening of short-term synaptic depression at *Dscamx3* NMJs became clearer when EJPs were evoked by trains of stimuli applied in a pattern that replicates burst firing of *Drosophila* motoneurons (Kauwe and Isacoff, 2013). At control NMJs, each train (10 stimuli at 10 Hz) caused a rapid decline in EJP amplitude of ~ 20% (Fig. 2B). The size of the first EJP of each of the 8 trains did not differ, indicating full recovery during the one minute interval between trains (Fig. 2C). The rapid decline and quick recovery are both in agreement with previous studies of *Drosophila* larval NMJs which describe fast depletion of an immediately releasable pool of vesicles and its fast replenishment (Delgado et al., 2000, Kauwe and Isacoff, 2013). At *Dscamx3* NMJs, the depression was enhanced to ~ 25% (Fig. 2B and C). It too was fast, robust and short-lived, as its magnitude was constant from train to train and there was full recovery of EJP amplitudes between trains (Fig. 2C). Burst firing of the nerve had no lasting effect on spontaneous transmitter release, as there was no difference in the distribution of mEJP intervals (*CSw*-, *P* = 0.2905; *Dscamx3*, *P* = 0.4628) or amplitudes (*CSw*-, *P* = 0.2796; *Dscamx3*, *P* = 0.9988) immediately before and after the series of trains (400 events; 50 from each of 8 recordings, recorded over ~ 20 s). Therefore, the extra copy of *Dscam* strengthened short-term synaptic depression of action potential-dependent vesicular release during high frequency firing of the nerve but did not change the speed of recovery.

### 3.3 A third copy of *Dscam* did not change the gross morphology of the NMJ

**Fig. 3:**
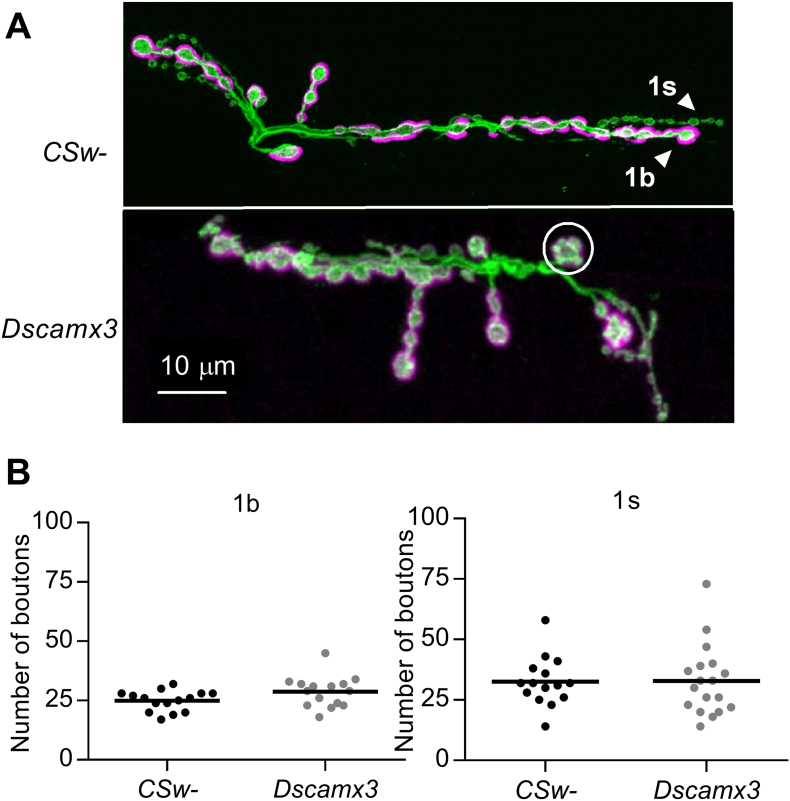
Bouton number is unchanged at *Dscamx3* larval NMJs. (A) Representative images of *CSw*- and *Dscamx3* larval NMJs at muscle 6/7 in abdominal section 2, stained for horseradish peroxidase (green) and Discs large (magenta). Arrowheads point to type 1 s (small) boutons (green) and type 1b (big) boutons (green and magenta). Circle indicates a satellite 1b bouton composed of a central bouton surrounded by multiple smaller boutons. (B) Comparison of the number of 1b (left) and 1s (right) boutons. There was no difference in the mean number between control and *Dscamx3* NMJs (1b boutons: *CSw*-, 24.93 ± 1.11, *N* = 15; *Dscamx3*, 28.8 ± 1.69, *N* = 15; *P* = 0.0883; 1s boutons: *CSw*-, 32.6 ± 2.62., *N* = 15; *Dscamx3*, 32.83 ± 3.42, *N* = 15; *P* = 0.786).

In adult *Dscamx3 Drosophila*, triplication of *Dscam* induces abnormal branching of sensory axons and impaired transfer of information along the neural circuit mediating touch perception (Cvetkovska et al., 2013), while a gain of function mutation results in enlargement of nerve terminals of C4 da neurons in *Drosophila* larvae (Kim et al., 2013). To investigate if altered synaptic transmission at *Dscamx3* larval NMJs was accompanied by changes in the gross structure of motor nerve terminals, NMJs at muscles 6/7 were stained with fluorescently-labelled antibodies for horseradish peroxidase (HRP) and Discs-Large (Dlg). The two motor neurons innervating the NMJ, one utilising type 1b (big) and the other type 1 s (small) boutons, were discriminated by bouton size and the greater expression of Dlg in the subsynaptic reticulum opposite 1b boutons (Menon et al., 2013). Analysis of stained NMJs revealed no difference between control and *Dscamx3* NMJs in the number of 1b or 1 s boutons (Fig. 3B) or the mean area of the muscle surface (*CSw*-, 87,771 ± 5307 μm^2^, *N* = 15; *Dscamx3*, 94,669 ± 4651 μm^2^, *N* = 15; *P* = 0.3367). A minority of *Dscamx3* NMJs (3/15) had one or two 1b satellite boutons, composed of several smaller boutons budding from a single larger central bouton (Menon et al., 2013), in contrast to the usual linear arrangement (Fig. 3A).

### 3.4 A third copy of *Dscam* produced a locomotor impairment

**Fig. 4:**
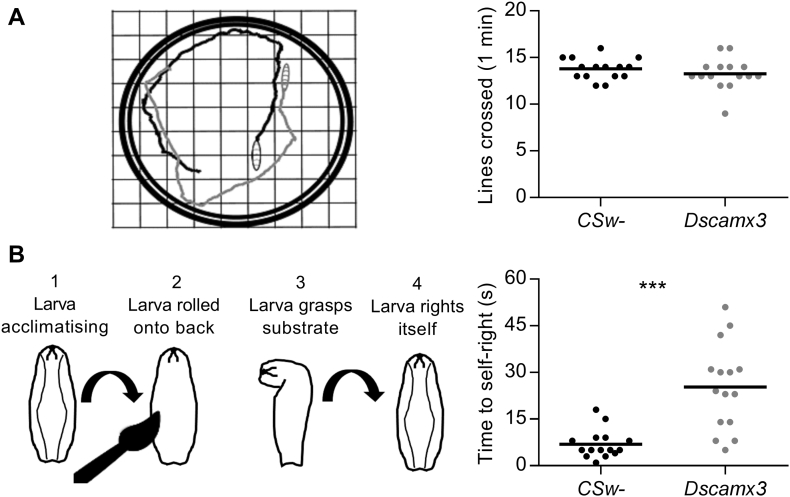
A third copy of *Dscam* slows self-righting behaviour. (A) Sample movement trajectories of *CSw*- (black) and *Dscamx3* (grey) third instar larvae. Number of lines (0.5 cm spacing) crossed did not differ (*CSw*-, 13.8 ± 0.27, *N* = 15; *Dscamx3*, 13.27 ± 0.43, *N* = 15; *P* = 0.323). (B) Sequence of movements performed by a larva during self-righting after being placed on its back with a fine brush. *Dscamx3* larvae took longer to complete the movements (*CSw*-, 6.87 ± 1.18 s, *N* = 15; *Dscamx3*, 25.27 ± 3.6 s, *N* = 15; *P* = 0.0002).

*Drosophila* larvae crawl by peristaltic muscle contractions that are driven by glutamate released from rhythmically firing motoneurons (Kohsaka et al., 2012). The possibility that the enhanced short-term synaptic depression of glutamate release at *Dscamx3* larval NMJs affected movement was assessed in a simple crawling assay. This revealed no effect on the distance travelled in 1 min of free movement (Fig. 4A). In contrast, performance was impaired in a self-righting assay (Fig. 4B), which is a more complex locomotor task during which a larva needs to perform a stereotyped sequence of movements to right itself after being rolled onto its back (Picao-Osorio et al., 2015). These results show that the extra copy of *Dscam* did not affect general fitness or the neural circuits that control peristaltic muscle contractions, but it did impair the correct operation of sensorimotor circuits that coordinate muscle contractions underlying more complex movements (Kohsaka et al., 2012).

## 4 Discussion

This study established that expression of a third copy of *Drosophila Dscam* altered glutamatergic synaptic transmission at the larval NMJ. The main presynaptically-mediated effects were stronger short-term depression of EJPs during burst firing of motoneurons and a rise in the frequency of spontaneous mEJPs. These changes were accompanied by a decrease in mEJP amplitudes. The extra copy of *Dscam* also impaired performance in a complex locomotor task.

Our findings that spontaneous transmitter release and short-term plasticity of evoked transmitter release were altered at *Dscamx3* larval NMJs indicate that overexpression of *Dscam* can modify glutamatergic synaptic transmission. *DSCAM* overexpression therefore may contribute to brain dysfunction in DS, since *DSCAM* levels are increased in DS brain (Alves-Sampaio et al., 2010, Saito et al., 2000) and mouse models of DS (Alves-Sampaio et al., 2010, Amano et al., 2004, Jia et al., 2011, Perez-Nunez et al., 2016). The increase in mEJP frequency, the absence of a change in EJP amplitude despite a decrease in mEJP size and stronger paired pulse depression, all suggest an increase in the basal probability of glutamate release. This is reminiscent of the elevated probability of glutamate release from cerebellar granule cells in the Ts65Dn mouse model of DS (Das et al., 2013), but enhanced transmitter released from these neurons is unlikely to be due to *Dscam* overexpression as human cerebellar granule cells do not express *DSCAM* (Saito et al., 2000). Our finding of a small but highly reproducible strengthening of the depression of EJP amplitude by high frequency stimuli trains suggests an enlargement in the size of the readily releasable pool of neurotransmitter vesicles (Kavalali, 2015), perhaps to facilitate an increase in quantal content of the EJP and thus maintain its amplitude, in response to the decrease in mEJP amplitude (Weyhersmuller et al., 2011). The modest slowing of the mEJP and EJP decays resembles the slowing of evoked EPSCs at cerebellar parallel fibre-Purkinje cell synapses of Tc1 mice (Galante et al., 2009), which could reflect *Dscam* overexpression in the parallel fibres or the Purkinje cells as *DSCAM* is known to be overexpressed in fibres of the cerebellar granule layer and in cerebellar Purkinje cells of DS brain (Saito et al., 2000).

Other previous studies of glutamatergic transmission in the Ts65Dn mouse model of DS have reported results that contradict our findings. They have found a reduction, and not the increase we observed, in the frequency of mEPSCs in hippocampal CA3 neurons (Hanson et al., 2007, Roncace et al., 2017), sEPSCs in neocortical neurons of Ts65Dn mice (Cramer et al., 2015) and sEPSCs in neurons derived from trisomy 21 induced pluripotent stem cells (Weick et al., 2013). Another study found speeding, and not the slowing we observed, of mEPSC waveform in cultured Ts65Dn hippocampal neurons (Best et al., 2008). The differences between the previous results and our findings could indicate that overexpression of *Dscam* alone does not cause the changes in synaptic function seen in DS mouse models and that triplication of other HSA21 gene orthologues is necessary. They could also indicate that synaptic function is differentially altered at different brain synapses in DS and that the effects at the glutamatergic synapse of *Drosophila* larvae model changes at brain synapses other than those in the hippocampus or neocortex. Moreover, a confounding factor in studies using Ts65Dn mice is the triplication of an additional ~ 35 protein coding genes which are not orthologous to HSA21 genes, but which may have modified the effects of the 90 HSA21 orthologues triplicated in Ts65Dn (Gupta et al., 2016). Also, Ts65Dn mice are trisomic for only ~ 55% of the HSA21 orthologues. Therefore, in order to better understand the changes in glutamatergic transmission in DS and their genetic basis, and to allow a comparison between our results using *Drosophila* with mouse models of DS, it would be informative to study synaptic function in the brain of multiple genetic mouse models of DS that do not have three copies of other, non-HSA21, orthologues.

The effects of *Dscam* overexpression on glutamatergic transmission that we observed occurred in the absence of gross changes in the structure of motor nerve terminals, as indicated by the unaffected numbers of boutons. This was unexpected as *Dscam* controls dendritic and axonal branching, and the precise apposition of presynaptic and postsynaptic elements, in mice and *Drosophila* (Alves-Sampaio et al., 2010, Andrews et al., 2008, Cvetkovska et al., 2013, Fuerst et al., 2009, Jain and Welshhans, 2016, Kim et al., 2013, Li et al., 2015). However, modifications in the fine structure of the NMJs cannot be excluded, as the lengthening of the synaptic events could reflect impeded clearance of glutamate from the synaptic cleft (Li et al., 2009). The effects on spontaneous transmission and short-term depression were relatively modest. Nevertheless, their sum impact across a population of synapses would be of sufficient magnitude to affect information processing. It is also possible that the effects on transmitter release are not limited to glutamatergic synapses but apply to the release of GABA from neurons such as GABAergic cerebellar Purkinje cells, which overexpress *DSCAM* in DS (Saito et al., 2000). This would affect motor function as Purkinje cells are the sole output of the cerebellar cortex (Apps and Garwicz, 2005).

The mechanisms by which the extra copy of *Dscam* affected multiple aspects of synaptic transmission remain to be elucidated. The effects could be due to a primary effect of *Dscam* overexpression in the motor nerve terminals or they could be compensatory responses to the smaller mEJP amplitude. In turn, the decrease in mEJP amplitude could reflect a lower vesicular concentration of glutamate or changes in the properties of the postsynaptic glutamate receptors, since both presynaptic and postsynaptic *Dscam* influences the arrangement of postsynaptic ionotropic glutamate receptors in *Aplysia* neurons (Li et al., 2009). We do not know if the effects were due to *Dscam* overexpression presynaptically, in the motor nerve terminals, or postsynaptically, in the muscle, or both. Previous studies have shown that *Drosophila Dscam* is expressed broadly in the developing and adult nervous system and localises to dendrites, axons and presynaptic terminals (Hummel et al., 2003, Hutchinson et al., 2014, Kim et al., 2013, Millard et al., 2010, Wang et al., 2004, Zhu et al., 2006); it has also been suggested to be expressed in larval muscle (Carrero-Martínez and Chiba, 2009). However, definitive mapping of the presence or absence of *Dscam* isoforms to presynaptic or postsynaptic elements of the larval NMJ is problematic due to extensive alternative splicing of *Dscam*, which consists of four clusters of 12, 48, 33 and 2 mutually exclusive exons that can form in excess of 38,000 protein isoforms, in a cell-specific manner (Schmucker, 2007).

The selective effect of *Dscam* overexpression on *Drosophila* neural circuits that control larval self-righting behaviour, without an effect on crawling speed, mirrors the more severe disruption of fine rather than gross motor skills of people with DS (Ferreira-Vasques and Lamônica, 2015, Marchal et al., 2016, Schott and Holfelder, 2015, Spanò et al., 1999). Likewise, a number of mouse models trisomic for regions of murine chromosomes orthologous to HSA21, which include *Dscam*, show no deficits in gross motor ability but are impaired in assays requiring balance and locomotor coordination (Belichenko et al., 2009, Goodliffe et al., 2016, Sago et al., 1998). Further evidence for the importance of the correct dosage of *Dscam* in determining optimal motor function is the disturbed locomotor coordination, despite largely spared walking ability, of *Dscam* loss-of-function mice (Lemieux et al., 2016, Thiry et al., 2016, Xu et al., 2011). In these mice, there is aberrant development of locomotor and sensorimotor circuits as well as enhanced depression of nerve-evoked potentials in skeletal muscle during high frequency stimulation of motor nerves, akin to the stronger synaptic depression we observed at *Dscamx3* larval NMJs (Lemieux et al., 2016). Similarly, *RNAi*-mediated knockdown of *Dscam* causes motor impairment in the beetle *T*. *castaneum* (Peuß et al., 2016), further indicating a conserved role across species. Altogether, these previous studies and our current study suggest that *DSCAM* overexpression in DS may contribute to motor disabilities experienced by people with DS.

## 5 Conclusions

This study shows that expression of a third copy of *Dscam*, a homologue of one of the genes on HSA21, was sufficient to modify synaptic function and disrupt locomotor performance in the model organism *Drosophila*. This novel evidence further elucidates the function of *DSCAM*. Further work is required to fully describe the role(s) of *DSCAM* overexpression in DS, particularly in the context of concomitant overexpression of other HSA21 genes.
